# Ibrutinib Plus R-ICE Induces Remission in Blastoid Variant Mantle Cell Lymphoma with CNS Relapse

**DOI:** 10.1155/2022/1930546

**Published:** 2022-05-06

**Authors:** Timothy S. Oh, Madelyn Burkart, Amir Behdad, Hatice Savas, Reem Karmali

**Affiliations:** ^1^Department of Medicine, Northwestern University Feinberg School of Medicine, Chicago, IL, USA; ^2^Division of Hematology/Oncology, Robert H. Lurie Comprehensive Cancer Center of Northwestern University, Northwestern University Feinberg School of Medicine, Chicago, IL, USA; ^3^Department of Pathology, Northwestern University Feinberg School of Medicine, Chicago, IL, USA; ^4^Department of Radiology, Northwestern University Feinberg School of Medicine, Chicago, IL, USA; ^5^Division of Hematology/Oncology, Robert H. Lurie Comprehensive Cancer Center of Northwestern University, Chicago, IL, USA

## Abstract

Mantle cell lymphoma (MCL) is an aggressive, difficult to treat subtype of lymphoma, resulting in relapses and poor outcomes. Novel agents such as Bruton tyrosine kinase (BTK) inhibitors have been studied in the treatment of relapsed/refractory (R/R) MCL. BTK inhibitor ibrutinib, in particular, has demonstrated improvement in survival outcomes of R/R MCL. Despite these advancements, many cases of MCL, including the more aggressive blastoid and pleomorphic variants, will undergo disease progression leading to poor survival outcomes. Blastoid variant MCL is associated with an increased risk of central nervous system (CNS) involvement, causing high mortality rates. In this case report, we discuss a patient with a diagnosis of blastoid MCL with CNS relapse who achieved a complete response (CR) after receiving standard rituximab plus ifosfamide-carboplatin-etoposide (R-ICE) salvage chemotherapy with the addition of ibrutinib. The patient subsequently underwent autologous stem cell transplantation (autoSCT) and maintained CR with ibrutinib maintenance.

## 1. Introduction

Mantle cell lymphoma (MCL) is an uncommon, aggressive subtype of non-Hodgkin lymphoma accounting for 3–10% of adult-onset lymphomas [1]. The common clinical course of MCL is characterized by recurrent relapses after initial therapy leading to poor survival outcomes, with a median survival of 4–5 years [2]. Current standard of care in fit patients broadly consists of three components: cytarabine-containing induction chemotherapy regimen followed by consolidation with autologous stem cell transplantation (autoSCT) and rituximab maintenance, while less fit patients receive less intensive therapy without autoSCT. There have been several novel agents studied for the treatment of relapsed/refractory (R/R) MCL including Bruton kinase (BTK) inhibitors. Ibrutinib, a first-generation BTK inhibitor, has shown notable improvement in survival outcomes in R/R MCL, with a 68% overall response rate when used as a single agent [3]. However, most patients will eventually progress, and survival remains poor for patients with high-risk disease features, including the aggressive morphologic variants (blastoid and pleomorphic variants), which comprise less than 20% of MCL cases [4].

Patients with blastoid variant MCL are at an increased risk for developing central nervous system (CNS) disease. CNS involvement in MCL is a rare, yet serious condition, and is known to have a high mortality rate with a median survival of 3.7 months [5]. It typically occurs later in the disease course during systemic relapse when treatment options are more limited. Prompt diagnosis and initiation of CNS-directed therapy is pertinent. We describe a case of blastoid MCL with CNS relapse demonstrating a complete response to treatment with the addition of ibrutinib to standard R-ICE salvage chemotherapy. Our patient was consolidated with an autoSCT and maintains response to ibrutinib maintenance therapy [6].

## 2. Case Presentation

**Figure 1:**
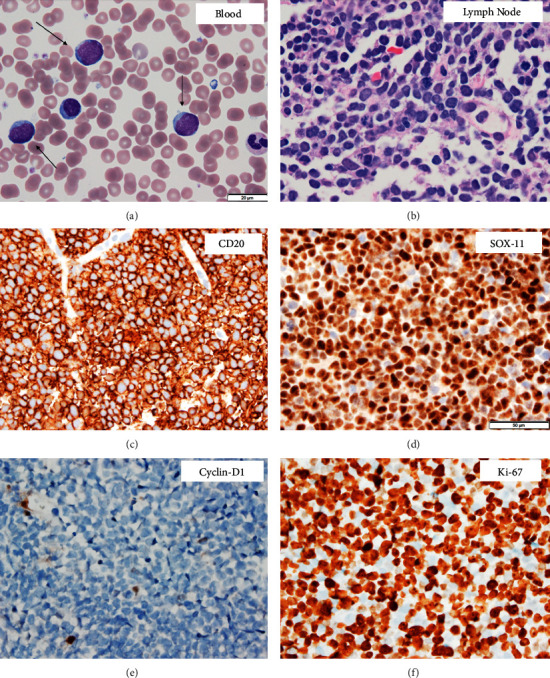
(a) Wright–Giemsa stain of peripheral blood smear shows circulating large atypical lymphoid cells, highlighted with arrows (1000×). (b) The hematoxylin and eosin section shows diffuse proliferation of large pleomorphic lymphoid cells in the cervical lymph node (600×). (c–f) Immunohistochemical stains (all 600×) demonstrate that the cervical lymph node neoplastic cells are positive for CD20 and SOX-11, while negative for cyclin-D1. Ki-67 highlights a very high proliferative index of ∼90%.

Our patient is a 60-year-old male with no significant past medical history who presented with three weeks of worsening left-sided abdominal pain and a large, mobile right neck mass. He had no B-symptoms on presentation. Laboratory evaluation was significant for leukocytosis of 59.3 (cells/L) with a differential of largely atypical lymphocytes (Figure 1(a)) and LDH elevated to 1246 (IU/L). CT neck/chest/abdomen/pelvis showed diffuse lymphadenopathy (LAD) with marked splenomegaly, concerning lymphoma. A right cervical lymph node biopsy revealed a diagnosis of blastoid variant MCL, further characterized as cyclin-D1 negative, SOX-11 positive, and p53 negative with a Ki-67 index of approximately 90% (Figure 1(b)–1(f)). Bone marrow biopsy revealed MCL, blastoid variant, with 70–80% involvement. Mantel cell lymphoma international prognostic index (MIPI) score was 7.5, consistent with high-risk disease.

**Figure 2:**
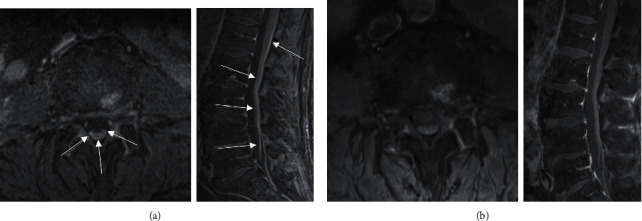
(a) Pretreatment axial and sagittal T1 weighted fat saturated postcontrast images show abnormal enhancement along the dorsal aspect of the spinal cord at the level of L1 and L2, with abnormal enhancement along the cauda equina nerve roots. Findings are highly suspicious for leptomeningeal disease with a differential of infectious and inflammatory etiologies. (b). Post-treatment axial and sagittal T1 weighted fat saturated postcontrast images show markedly improved enhancement at the level of L1 and L2 and of the conus medullaris and cauda equina nerve roots, with very subtle minimal enhancement.

Staging PET/CT revealed diffuse hypermetabolic LAD above and below the diaphragm as well as known splenomegaly. The patient was treated with 6 cycles of rituximab plus cyclophosphamide, vincristine, doxorubicin, and dexamethasone (R-HyperCVAD) with intrathecal (IT) methotrexate (MTX). After 4 cycles, interim bone marrow biopsy showed no evidence of disease and interim PET/CT was consistent with complete response (CR). An autologous SCT after completion of induction chemotherapy was planned. However, 6 weeks after completion of therapy, the patient began experiencing double vision and facial paresthesias. A lumbar puncture was performed and showed elevated protein (>200 mg/dL) with negative cytometry and cytopathology for lymphoma. An MRI brain/orbits did not demonstrate signs of leptomeningeal disease or obvious abnormality to explain symptoms, but an MRI of the spine showed lumbar enhancement concerning disease involvement (Figure 2(a)). Two repeat cerebrospinal fluid (CSF) evaluations were similar to prior results, demonstrating elevated protein but negative cytometry and cytopathology. An autoimmune and infectious workup was negative. Given the CSF results and concerning MRI findings with known high-risk blastoid MCL, the patient was treated for presumed secondary CNS involvement with the addition of ibrutinib 560 mg daily to CNS-directed salvage chemotherapy with three cycles of R-ICE with IT MTX. The only complication of salvage therapy was IT MTX-induced myelopathy noted on the MRI spine. MTX was discontinued, and the patient completed a dexamethasone taper along with memantine with improvement in symptoms. No toxicities related to ibrutinib were noted. Repeat MRI spine after completion of therapy demonstrated CR (Figure 2(b)) with the resolution of symptoms and repeat PET/CT imaging showed CR2.

The patient underwent autoSCT with myeloablative busulfan and thiotepa conditioning after achieving a CR2. Ibrutinib was held during the peritransplant period. Post-transplant course was complicated by prolonged pancytopenia secondary to chemotherapy, for which the patient received granulocyte colony-stimulating factor (G-CSF) to assist with count recovery as well as intravenous immunoglobulin (IVIG).

Ibrutinib was resumed on day 46 post-transplant after count recovery. Reimaging on day 95 showed CR, and no enhancement was noted on MRI spine imaging. Currently, the patient continues ibrutinib 560 mg daily without any dose adjustments and has tolerated this well, with the only complication of ibrutinib being thrombocytopenia. The patient is now 31 months post-transplant and continues to be in CR.

## 3. Discussion

Management of relapsing CNS disease in MCL patients remains a challenging task. Currently, there is no consensus on standard treatment. Ibrutinib, however, has been a key focus in treatment and has been shown to be efficacious as a single-agent therapy. A 2013 phase 2 study, for example, demonstrated a response rate of 68%, estimated median response duration of 17.5 months, and estimated median progression-free survival (PFS) of 13.9 months in R/R MCL [3]. Of particular importance is the ability of ibrutinib to cross the blood-brain barrier to target CNS relapse, as demonstrated in prior pharmacokinetic studies [7].

Prior case reports have also shown promising outcomes by using ibrutinib in relapsing MCL patients with CNS involvement. A 2015 report of 3 patients with symptomatic CNS relapse showed rapid response after initiating ibrutinib as a single agent at a standard dose of 560 mg/d [8]. Clinical response times were noted after 3 days to 1 week after initiation of therapy. Two of the patients achieved CR at 9 months to 1 year while the third patient showed partial response (PR) at 2 months. A 2017 report of a patient with symptomatic CNS relapse also showed promising results with ibrutinib monotherapy (dose reduced to 280 mg/d in the setting of recurrent atrial fibrillation and concurrent treatment with amiodarone), sustaining CR 13 months after initiation of therapy [9].

More recently, a 2020 report of 84 patients compared outcomes between patients who received standard immune-chemotherapy (rituximab combined with either high-dose MTX, bendamustine, IT chemotherapy, or radiation therapy) versus ibrutinib alone for MCL with CNS relapse [10]. This is the first large cohort study of its kind examining the effectiveness of ibrutinib as a single agent in CNS relapse of MCL and showed improved overall response rate and CR rate (17% in the standard group vs 42% in the ibrutinib group) [10]. The ibrutinib group also showed improved 1-year overall survival (OS) compared to the standard cohort (61% vs 16%) [10]. It is evident that ibrutinib continues to act as a pivotal component of MCL with CNS relapse.

The outcome of the patient discussed in this case is particularly noteworthy given his multiple high-risk disease characteristics, including blastoid variant, high Ki-67 index, and high MIPI score. Also, of particular interest is the fact that the patient has sustained a CR for over two years after completing salvage R-ICE combined with ibrutinib followed by an autologous SCT and ibrutinib maintenance, a combination which has not been reported elsewhere in the current literature specifically for R/R MCL. A phase I study demonstrated that ibrutinib combined with R-ICE for salvage therapy in R/R diffuse large B-cell lymphoma (DLBCL) had an overall response rate of 90% while being well-tolerated with low toxicity rates [6]. As seen in our patient, the translation of this approach with the addition of ibrutinib to R-ICE represents a promising new salvage chemotherapy regimen for relapsing MCL patients including those with CNS involvement. The addition of ibrutinib to R-ICE as salvage therapy and maintenance post autoSCT showed an impressive effectiveness in maintaining CR and preventing relapse in a patient with high-risk disease involving the CNS with an otherwise dismal prognosis. Our experience provides rationale for the evaluation of this approach on a larger scale in a clinical trial which may verify this approach as a promising new standard therapy in an otherwise challenging disease.
